# The Impact of Mentorship on Female Plastic Surgeons in Canada: A Cross-Sectional Survey

**DOI:** 10.1177/22925503261479647

**Published:** 2026-08-31

**Authors:** Elly Dimya Htite, Caroline Esmonde-White, Rawan ElAbd, Sabrina Cugno

**Affiliations:** 1 Faculty of Medicine and Health Science, 7512 McGill University, Montreal, Quebec, Canada; 2 Division of Plastic and Reconstructive Surgery, 55980 McGill University Health Center, Montreal, Quebec, Canada

**Keywords:** mentorship, plastic surgery, women physicians, surgical education, burnout, work-life balance, mentorat, chirurgie plastique, femmes médecins, formation chirurgicale, épuisement professionnel, équilibre travail-vie personnelle.

## Abstract

**Introduction:**

Mentorship is important for professional development, reducing burnout, and promoting diversity within surgical programs. Notably, mentorship has been established as a tool for encouraging women to pursue surgical careers. In Canada, female plastic surgeons make up a third of surgeons. However, no study to date has investigated the effect of mentorship on female plastic surgeons in Canada.

**Methods:**

We conducted a cross-sectional study assessing the impact of mentor-mentee relationships of female plastic surgeons across Canada. An online questionnaire was sent to female plastic surgeons across 13 Canadian academic institutions. The survey included mentee and mentor demographics, mentee-mentor relationship, and mentorship perspectives.

**Results:**

Twenty-five participants completed the survey with a mean age of 33.2 (SD ± 9.4) from 10 academic institutions. 72% (n = 18) were residents and 28% (n = 7) were consultants. 60% (n = 15) had a mentor, with 40% (n = 6) having more than three mentors; 60% (n = 9) of mentors were women and 30% (n = 6) were men. Most participants met mentors through research (33%; n = 5) and formal mentorship programs (33%; n = 5). Main topics discussed include academic proclivity (80%; n = 12), surgical skills (80%; n = 12), and work-life balance (80%; n = 12). Main mentor contributions include development of surgical (93%; n = 14) and clinical skills (80%; n = 12), pursuit of plastic surgery (80%; n = 12), and research (73%; n = 11).

**Conclusion:**

Mentorship plays an instrumental role for female plastic surgeons in developing clinical and surgical skills and providing encouragement to pursue a career in plastic surgery. This investigation identifies strengths and gaps in mentor-mentee relationships that can promote future effective mentorship and diversity in plastic surgery.

## Résumé

**Introduction:**

Le mentorat revêt de l’importance pour le perfectionnement professionnel, la réduction de l’épuisement professionnel et la promotion de la diversité au sein des programmes de chirurgie. Il est notamment établi qu’il favorise l’orientation des femmes vers une carrière en chirurgie. Au Canada, les chirurgiennes plasticiennes représentent le tiers des spécialistes en chirurgie. Cependant, aucune étude n’a encore porté sur l’effet du mentorat au sein de cette population.

**Méthodes:**

Les chercheuses ont réalisé une étude transversale sur les effets des relations mentor-mentorée chez les chirurgiennes plasticiennes du Canada. Ainsi, les chirurgiennes plasticiennes de treize établissements universitaires canadiens ont reçu un questionnaire en ligne qui portait sur les données démographiques des mentorées et des mentors, la relation mentor-mentorée et les points de vue sur le mentorat.

**Résultats:**

Au total, 25 participantes, d’un âge moyen de 33,2 ans (ÉT ± 9,4) et provenant de dix établissements universitaires, ont rempli le sondage. De ce nombre, 72 % (n=18) étaient résidentes et 28 % (n=7), consultantes. Des 60 % (n=15) qui comptaient sur un mentor, 40 % (n=6) en avaient plus de trois. De plus, 60 % (n=9) des mentors étaient des femmes et 30 % (n=6), des hommes. La plupart des participantes ont rencontré leur mentor dans le cadre de programmes de recherche (33 %; n=5) et de mentorat (33 %; n=5) structurés. Les échanges portaient surtout sur les aptitudes scientifiques (80 %; n=12), les compétences chirurgicales (80 %; n=12) et l’équilibre entre le travail et la vie personnelle (80 %; n=12). Pour ce qui est des principales contributions des mentors, elles incluaient l’acquisition de compétences en chirurgie (93 %; n=14) et en clinique (80 %; n=12), la poursuite d’une carrière en chirurgie plastique (80 %; n=12) et la recherche (73 %; n=11).

**Conclusion:**

Le mentorat joue un rôle déterminant dans l’acquisition des compétences des chirurgiennes plasticiennes en clinique et en chirurgie, en plus de les encourager à poursuivre une carrière en chirurgie plastique. La présente étude décrit les forces et les lacunes des relations mentor-mentoré afin de favoriser un mentorat efficace et la diversité en chirurgie plastique.

## Background

Mentorship is a critical tool for professional development in all disciplines of medicine.^1^ Ongoing and adaptive mentorship becomes important in surgical careers due to the evolving role of the surgeon throughout their training.^2^ Mentorship has been linked not only to professional development but also higher job satisfaction, greater program diversity, and protection against physician burnout.^3,4^ In a prior study analyzing the careers of top academic plastic surgeons, strong mentorship was emphasized as an influential factor.^5^ Even within medical school, students who had resident or faculty mentors were more likely to match into plastic surgery programs compared to students who did not.^6^ In a study of medical students who recently matched to plastic surgery, 80% cited that mentoring relationships had impacted their decision to pursue the specialty.^7^ Students have also stated that strong mentor-mentee relationships were a contributing factor when ranking different plastic surgery residency programs.^8^

Mentorship is especially important for women pursuing plastic surgery. Despite current statistics demonstrating gender parity in newly admitted medical students,^9^ only 34% of current practicing surgeons in Canada are women.^10^ Mentorship has been shown to be an effective method to promote women in plastic surgery. Mentors’ ability to attract diverse groups of medical students has been shown to reduce gender and racial disparities in plastic surgery residency programs.^11^ When investigating the diversity of plastic surgery residency programs in the United States, programs with female plastic surgery chairs had a 45% increased likelihood of having female plastic surgery residents.^12^ Additionally, female medical students named female role models as an influence in their decision to pursue a career in plastic surgery.^13^

Despite these advancements in equality and diversity, many barriers remain, particularly in relation to mentorship. Across all disciplines of surgery, female medical students cited lack of mentorship as a limitation in pursuing surgical careers.^14^ Further, women pursuing plastic surgery were less likely to report strong mentor relationships compared to their male counterparts.^15^ The ongoing benefit of mentorship has caused formal mentorship programs to be developed for women in surgery. Some of these organizations, such as the Association of Women Surgeons, work to “inspire, encourage and enable women surgeons to realize their professional and personal goals.”^16^ These goals are echoed in more focused groups, including the Women Plastic Surgeon of Canada Group, which promotes and supports women while they pursue successful careers in plastic surgery.^17^

Although mentorship is recognized as an important tool for professional and personal development, no studies have investigated the effect of mentorship on female plastic surgeons worldwide or in Canada. In this study, we aim to assess the impact of mentor-mentee relationships on the pursuit of plastic surgery and the perceived benefits. Through understanding the role mentorship plays for women in the field of plastic surgery in Canada, we aim to identify aspects of such relationships to encourage future effective and meaningful mentor-mentee relationships.

## Methods

This study utilized a cross-sectional survey sent to female plastic surgery residents and attendings across Canadian academic institutions, encompassing mostly academic or institution-affiliated residents and attendings. The survey was designed using SurveyMonkey, with the link being emailed to the administration of each academic institution. Prior to starting the survey, participants were made aware that survey completion was voluntary and anonymous. The survey was developed based on two published studies assessing mentorship in plastic surgery residents in the United States.^7,18^ This study was approved by the Institutional Review Board of McGill University. The survey is organized in four categories: (1) Mentee demographics, (2) Mentor demographics, (3) Mentee-mentor relationship, and (4) Mentorship perspective. The full survey is available in Supplementary Appendix 1. Data were collected over a period of 12 weeks, with one follow-up email sent to remind participants to complete the survey.

Survey responses were exported and analyzed using Microsoft Excel. Quantitative data were expressed as mean ± SD. Categorical data were expressed as frequency and percentage. A thematic analysis was performed for open-ended questions in which major themes were identified and summarized.

## Results

A total of 31 participants responded to the survey. Based on the Canadian Society for Plastic Surgeons, there is an estimated 90 out of 438 members who identify as female, with an additional 62 female identifying plastic surgery residents.^17^ With a total of 152 female plastic surgery attending and resident doctors in Canada, the survey had a completion rate of 20.3%.^17^ Of the participants, four had to be excluded due to survey incompletion, and two were excluded as they identified as male. As such, a total of 25 participants were analyzed in our study. The average age of participants was 33.2 ± 9.4 years. The majority of participants (72.0%, n = 18) were residents in plastic surgery, while the remaining participants were attending surgeons (28.0%, n = 7). Remaining demographic information is summarized in Table 1.

**Table 1.** Mentee Demographics.

Demographic Data	_	Participants (n = 25)
Gender	Female	100% (25)
Age		33.2 ± 9.4
Ethnicity	Caucasian/White	60.0% (15)
	Asian/Polynesian	16.0% (4)
	Arab/North African	16.0% (4)
	Mixed	8.0% (2)
Position	Resident	72.0% (18)
	Attending	28.0% (7)
Fellowship trained	Yes	28.0% (7)
	No	72.0% (18)

Of the participants, 60.0% (n = 15) stated having at least one mentor in plastic surgery. Of those, 40.0% (n = 6) had more than three mentors throughout their training. In terms of mentor demographics, the majority were women (66.7%, n = 10), white/Caucasian (86.7%, n = 13), attending physicians (86.7%, n = 13), and in their 30s (46.7%, n = 7) or 40s (40.0%, n = 6). Additional mentor demographics are summarized in Table 2.

**Table 2.** Mentor Demographics.

Demographic Data	_	Participants (n = 15)
Gender	Female	60.0% (9)
	Male	40.0% (6)
Age	30-39	46.7% (7)
	40-49	40.0% (6)
	50-59	13.3% (2)
Ethnicity	Caucasian/White	86.7% (13)
	Arab/North African	13.3% (2)
Position	Resident	6.7% (1)
	Attending	86.7% (13)
	Fellow	6.7% (1)
Fellowship trained	Yes	86.7% (13)
	No	13.3% (2)

**Figure 1.**
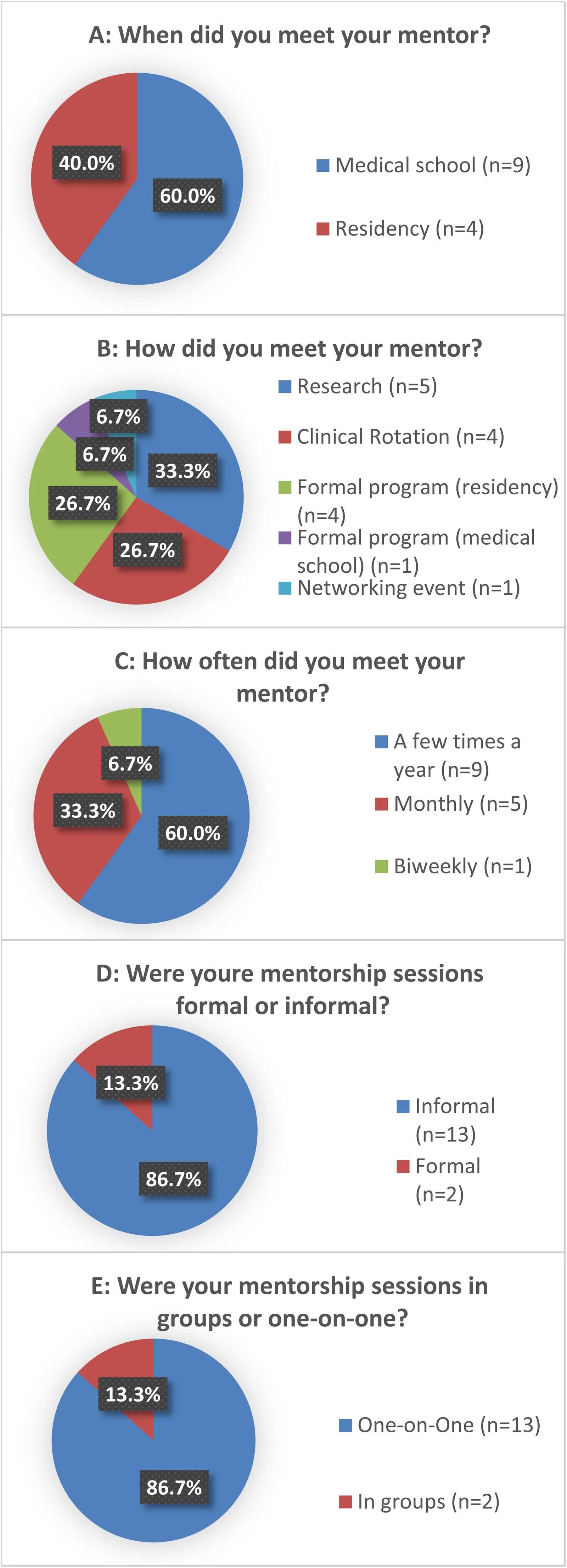
Summarized mentorship relationships between mentor and mentees. Includes when mentees met their mentors (A), how they met their mentors (B), how often they met with their mentors (C), if mentorship sessions were formal or informal (D), and if sessions were in group or one-on-one settings (E).

The majority (60.0%, n = 9) of participants had met their mentor in medical school, while 40.0% (n = 6) met their mentors during their residency training. Participants met their mentors most commonly through research experience (33.3%, n = 5), but other settings include clinical rotations (26.7%, n = 4), formal mentorship program during residency (26.7%, n = 4), formal mentorship program during medical school (6.7%, n = 1), and networking events (6.7%, n = 1). The majority of mentorship sessions were informal (86.7%, n = 13), occurred in one-on-one groups (86.7%, n = 13), and occurred a few times a year (60.0%, n = 9). The data are summarized in Figure 1.

**Figure 2.**
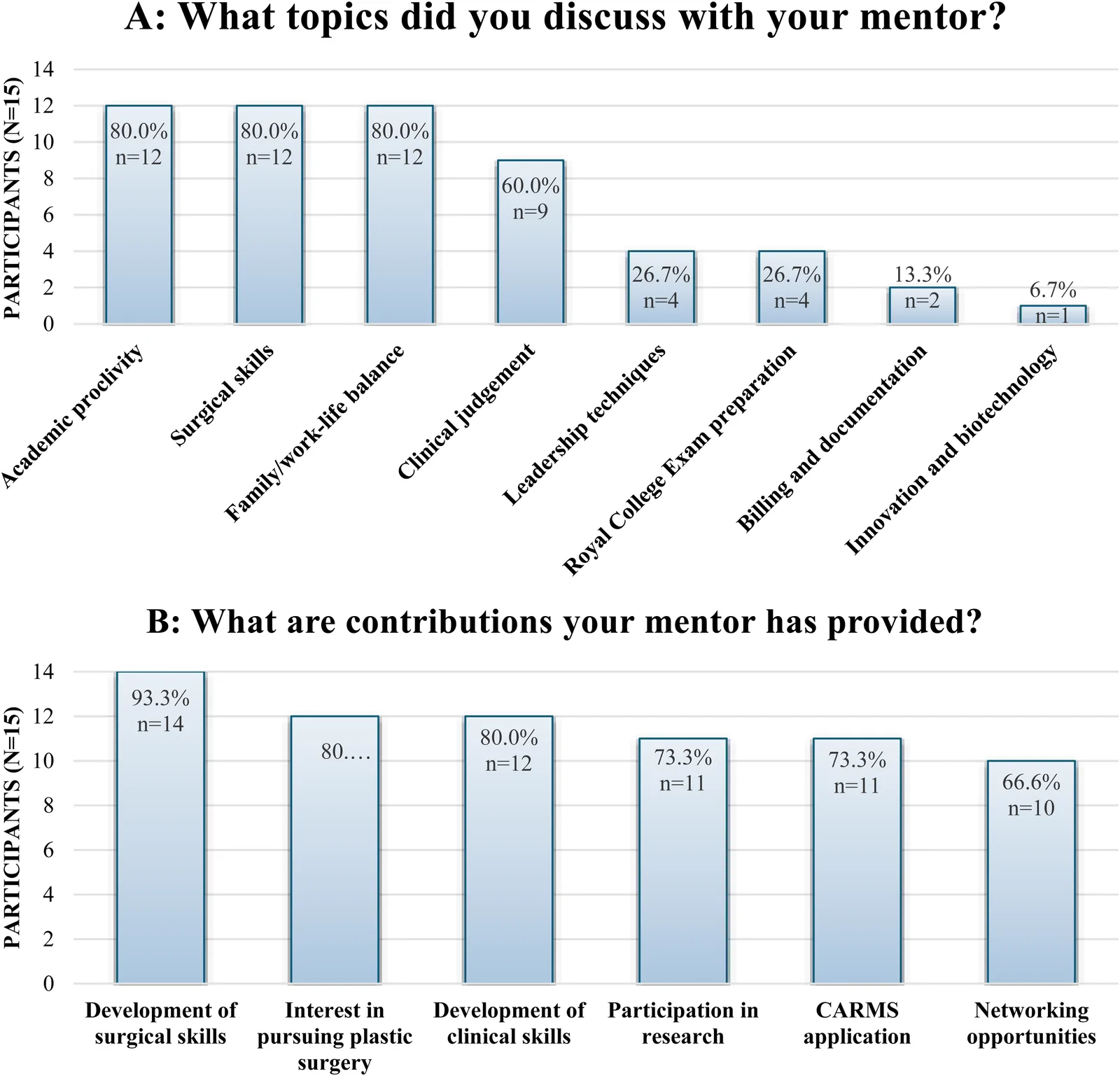
Topics discussed (A) and mentorship contributions (B) provided to mentees throughout their mentor-mentee relationships.

Topics discussed during mentorship sessions include academic proclivity (80.0%, n = 12), surgical skills (80.0%, n = 12), managing family/work-life balance (80.0%, n = 12), clinical judgment, (60.0%, n = 9), leadership techniques (26.7%, n = 4), preparation for the Royal College exam (26.7%, n = 4), billing and documentation (13.3%, n = 2), and innovation and biotechnology (6.7%, n = 1). When asked about contributions from their mentors, participants cited the development of surgical skills (93.3%, n = 14), interest in pursuing plastic surgery (80.0%, n = 12), development of clinical skills (80.0%, n = 12), participation in research opportunities (73.3%, n = 11), Canadian Resident Matching Service applications (73.3%, n = 11), and networking opportunities (66.7%, n = 10) (Figure 2).

**Figure 3.**
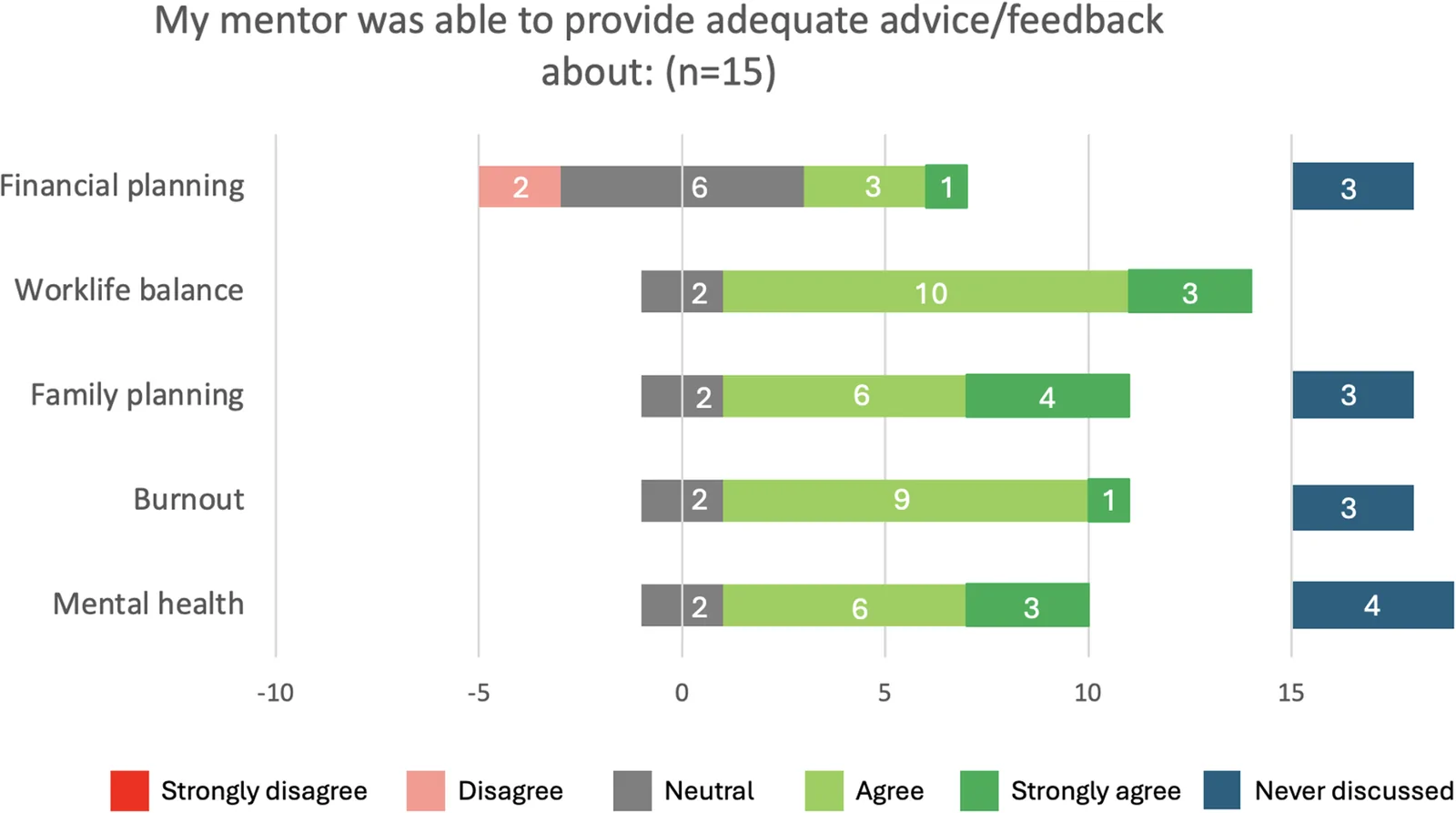
Mentorship perspectives on the quality of advice and feedback provided to mentees.

Perspective of the quality of mentorship was asked to participants who declared having a mentor (n = 15). Of those, the majority believed adequate advice was given regarding work-life balance (86.7%, n = 13), family planning (66.7%, n = 10), burnout (66.7%, n = 10) and mental health (60.0%, n = 9). However, some mentees believed they were given neutral (40.0%, n = 6) or inadequate (13.3%, n = 2) advice on financial planning. Of note, 26.7% (n = 4) of mentors did not discuss mental health, and 20.0% (n = 3) did not discuss burnout, family planning, or financial planning (Figure 3).

When asked, the majority of participants described their most meaningful contributions from their mentors to be related to guidance, support, and encouragement in their pursuit of plastic surgery (60.0%, n = 9). Providing networking opportunities (26.7%, n = 4) was also cited as an important contribution. When asked about areas of improvement, participants cited more help with research (6.7%, n = 1), networking opportunities (6.7%, n = 1), career planning (6.7%, n = 1), surgical skills (6.7%, n = 1), and reaching out for more frequent mentoring sessions (6.7%, n = 1).

**Figure 4.**
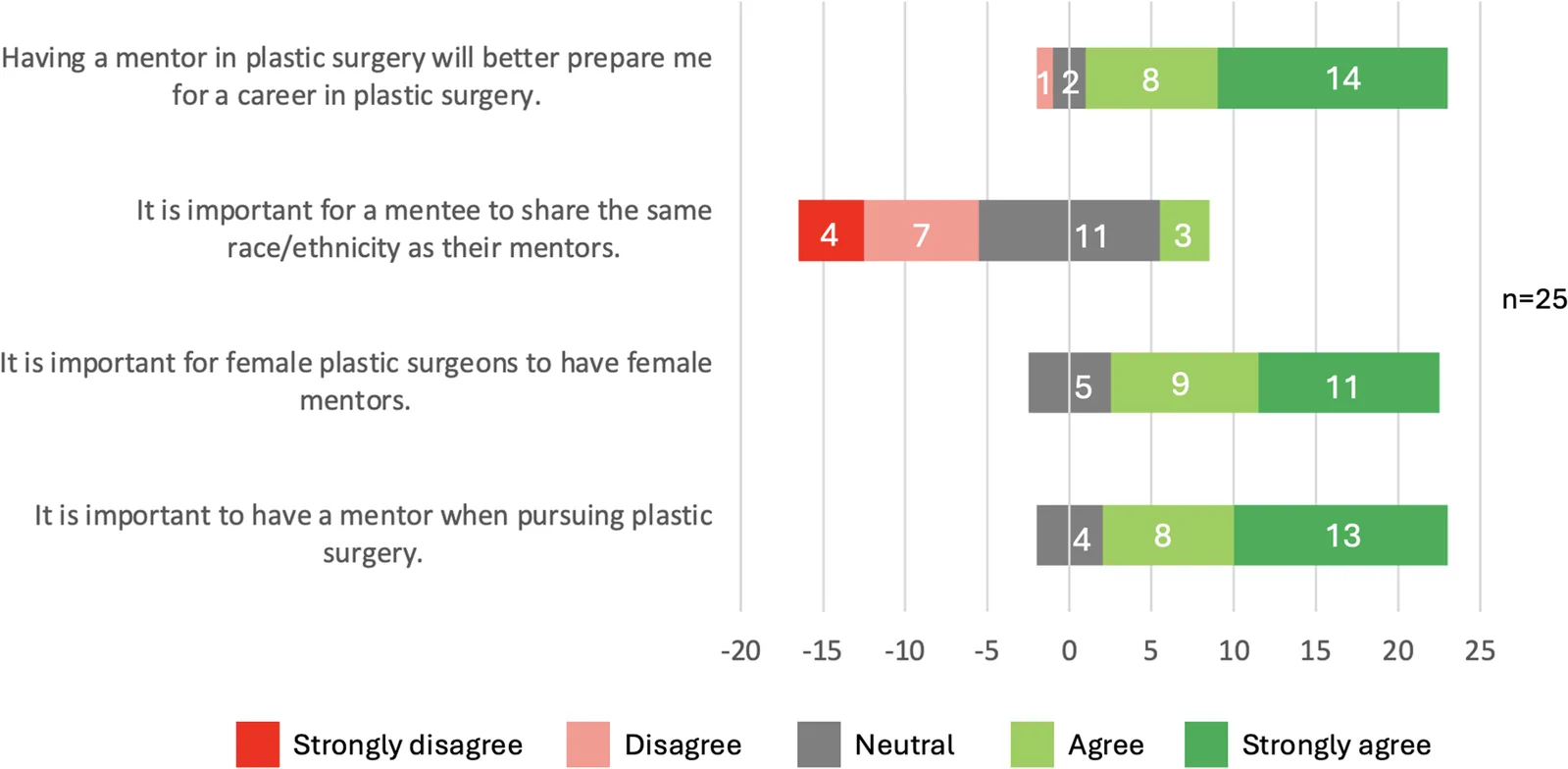
Global mentorship perspectives of the importance of mentorship in the pursuit of a career in plastic Surgery.

All participants (n = 25) were asked about their global perspectives on mentorship in the pursuit of a career in plastic surgery. The majority believed having a mentor better prepares someone to pursue a career in plastic surgery (88.0%, n = 22), that it is important to have a mentor when pursuing plastic surgery (84.0%, n = 21), and that it is important for female plastic surgeons to have female mentors (80.0%, n = 20). However, 44.0% of participants (n = 11) disagreed that it was important for mentors and mentees to share the same race and/or ethnicity (Figure 4).

## Discussion

### Current State of Mentorship Among Female Plastic Surgeons

It is previously well-established that mentorship is important in the pursuit of a surgical career. Prior studies have demonstrated both the importance of mentorship in the pursuit of the specialty in general^7,8^ as well as the impact of female role models in female medical students’ decisions to choose plastic surgery training.^12,13^ However, no previous study to date has measured the impact of mentorship on female plastic surgeons. In this study, we demonstrate the prevalence of mentorship relationships in women who have successfully pursued a career in plastic surgery in Canada and the impact on professional development. Participants in this study cited significant positive impact from their mentors, including exposure to research opportunities, networking, development of surgical skills, and academic productivity.

Of importance, the majority of study participants (60.0%, n = 15) reported that their mentors were mostly female (60.0%, n = 9). Given that only an estimated 27% to 37.0% of all plastic surgeons are female within North America,^19,20^ our study confirms that a significant proportion of female plastic surgeons are specifically involved in mentoring female mentees. This poses additional responsibilities on female plastic surgeon outside of typical clinical and academic involvement, an example of the minority tax. The minority tax is a concept seen within medicine, in which individuals who identify as part of a minority burden additional responsibilities to promote equity and diversity initiatives within their respective fields.^21^ This includes dedication to mentorship, which is often seen as a responsibility of minority physicians to be involved in.^22^ While many find purpose and understand the importance of strong mentorship, the additional expectations of mentorship involvement may provide to overall physician burnout in already well-known minority groups.

Interestingly, the majority of participants agreed that a same gender mentor is important while sharing other demographic factors, such as ethnicity and race, were deemed less important (Figure 4). Given the shared unique experiences of female plastic surgeons in a male-dominated field, participants acknowledged the role of same-sex mentorship in motivating the next generation of women surgeons. However, it would be remiss not to acknowledge the role of male mentors, as 40.0% of participants stated that they had meaningful and important relationships with male mentors who also had a tremendous positive impact on their careers.

### Importance of Mentorship for Female Plastic Surgeons

Female mentorship extends beyond the bounds of plastic surgery. In a 2021 study, South African women working in the public sector cited mentorship to be valuable in their career advancement, regardless of the gender of their mentors.^23^ Mentorship is cited as a critical tool for promoting women in leadership positions in global health, which remains vital as women face unique burdens of disease whose interests are best represented when women are involved in policy decision-making.^24^ Even within medicine, mentorship remains vital in promoting and retaining female physicians within positions in academic medicine.^25^

Although mentorship is known to decrease racial and gender diversities within surgical training programs,^11^ the nature and substance of these relationships, including what is being discussed, exchanged, and how it is shaping trainee's experiences, has not been well-documented. In this study, we reveal the nature of these relationships being largely curriculum-driven and focused on work-oriented topics. This is contrary to the expectation of relationships centering on unique experiences for women and underrepresented minorities such as navigating gender inequalities, work-life balance, family planning, or mental health. This suggests that these topics are being navigated elsewhere, perhaps through peer-to-peer mentorship.

While promising, previous studies suggest a lack of female mentorship in plastic surgery when compared to their male counterparts.^15^ This is particularly significant as female surgeons have unique challenges. For example, family planning as a female in a surgical specialty during childbearing years has been garnering increasing attention in recent years.^26^

### Improvements in the Current State of Mentorship

Our data show that the majority of mentees met with their mentor several times per year (60.0%, n = 9). Given the minority of female representation of Canadian surgeons (34%),^10^ while 60.0% of participants identified having female mentors, we can presume that mentors share their time among multiple mentees in an academic setting. This could theoretically provide a higher time burden to each female mentor, having to provide more mentorship sessions to multiple mentees to maintain this discrepancy.

When asked, participants identified areas of improvement with their current mentor-mentee relationships, including more frequent mentoring sessions. Open communication between parties helps to identify the unique needs and expectations of mentees while balancing the capacity of mentors. Such communications are encouraged between mentors and mentees, to ensure both can obtain the most of their relationships.

When assessing the quality of advice from mentees (Figure 3), more social concepts regarding wellness, such as mental health, burnout, and family planning, were never discussed between mentors and mentees. This may represent a limitation of current mentorship practices, which tend to focus on career advancements rather than holistic professional development. This can make longevity of successful careers in plastic surgery more challenging. In a 2012 survey of attending plastic surgeons, 40.0% of staff reported leaving academic positions within 5 years of practice, attributing lack of mentorship and burnout as contributory to their departure.^27^ Given that female plastic surgeons already represent a minority in academic positions,^28^ redefining the mentor-mentee relationship as being a fundamental component of physician wellness rather than a tool for academic development may provide further social resources to aid aspiring plastic surgeons throughout their training.

### Future of Mentorship

There is a need for further longitudinal research on the lasting effects of mentorship for female plastic surgeons. Longitudinal research would identify gaps in current mentor-mentee relationships to improve the quality of future mentor-mentee pairing, especially as part of a formal mentorship program, such as with the 33.3% of our participants who met a mentor through a formal program in our study. Secondly, it would provide insight on the lasting impact of mentorship early in someone's career and to assess how it shapes trainees as they become attendings and take on the role of mentor for future mentees. Lastly, longitudinal studies are needed to better inform the cyclical nature of mentor-mentee relationships. Early career mentees ultimately become mentors to the next generation of aspiring surgeons, and their own mentors provide insight of how they subsequently provide mentorship in the future. Understanding the quality of their mentorship when they were mentees, and how it translates to their ability to mentor, would shed insight on how to improve gender disparities and promote women within academic and leadership positions within plastic surgery.

## Conclusion

Our study is the first to assess the quality of mentor-mentee relationship of female plastic surgeons in Canada. Our data suggest that mentorship plays a critical role for female plastic surgeons in the development of clinical and surgical skills, as well as provide opportunities for career development. Further, mentorship provides encouragement to mentees wishing to pursue a career in a male-dominated field, as many participants report meetings mentors early in their training trajectory. However, further investigations are needed to identify gaps in mentor-mentee relationship to promote effective mentorship. Longitudinal studies may provide more insight to assess the evolving nature of mentorship and how specific periods in one's career may benefit from mentorship. Ultimately, mentorship remains a crucial tool to promote effective mentorship and diversity in the field of plastic surgery.

## Supplemental Material

sj-docx-1-psg-10.1177_22925503261479647 - Supplemental material for The Impact of Mentorship on Female Plastic Surgeons in Canada: A Cross-Sectional Survey Supplemental material, sj-docx-1-psg-10.1177_22925503261479647 for The Impact of Mentorship on Female Plastic Surgeons in Canada: A Cross-Sectional Survey by Elly Dimya Htite, Caroline Esmonde-White, Rawan ElAbd and Sabrina Cugno in Plastic Surgery
