# Supplementary material for: The Impact of Mentorship on Female Plastic Surgeons in Canada: A Cross-Sectional Survey
Source: Plast Surg (Oakv). 2026 Aug 31:22925503261479647. Online ahead of print. doi: 10.1177/22925503261479647 (PMC13530041; doi:10.1177/22925503261479647)
Supplement: Supplementary file 1 [file sj-docx-1-psg-10.1177_22925503261479647.docx]

**The Impact of Mentorship on Female Plastic Surgeons in Canada: A Cross-Sectional Survey: Appendix**

**APPENDIX 1: Survey sent to study participants**

Mentee demographics:

- What is your age?
- What is your race/ethnicity?
- What gender do you identify most as?
- What medical school did you graduate from?
- What year did you graduate from medical school?
- What is your current level of training? (resident, fellow, attending)
- Where did you do your residency?
- If applicable, what fellowship are you currently participating in or have completed? At which institution?
- During your career, have you had a mentor in plastic surgery?

Mentor demographics:

- How old (approx.) was your mentor when they became your mentor?
- Which gender does your mentor identify most with?
- What was your mentor’s race/ethnicity?
- What was your mentor’s level of training when they were your mentor?
- What medical school did your mentor graduate from?
- Where did your mentor go for their residency?
- Did your mentor participate or complete a fellowship? If yes, what fellowship?

Mentee-mentorship relationship:

- How many mentors have you had in your career?
- At what stage of your career did you meet your mentor?
- How did you meet your mentor?
  - Formal mentor program through medical school
  - Formal program through residency program
  - Formal program through national organization
  - Mutual friends/families
  - Clinical rotation
  - Research experience
  - Conference/meeting
  - Others (please specify)
- How often would you meet with your mentor?
- How would you communicate with your mentor (in person, phone, email, text message, other)
- Were your mentorship sessions formal or informal?
- Would you meet your mentor one-on-one or in groups with other mentees?
- Which of the following topics were discussed with your mentor?
  - Academic development
  - Clinical judgment
  - Surgical skill development
  - Managing family/work-life balance
  - Billing and documentation
  - Leadership techniques
  - Royal College Exam preparation
  - Innovation and biotechnology
- Did your mentor contribute to the following:
  - Interest in pursuing plastic surgery
  - Participation in research
  - Networking opportunities
  - CARMS application
  - Development of clinical skills
  - Development of surgical skills

Mentorship perspective

- My mentor was able to provide adequate advice/feedback about mental health
  - Strongly agree
  - Agree
  - Neutral
  - Disagree
  - Strongly disagree
  - Never discussed
- My mentor was able to provide adequate advice/feedback about burnout
  - Strongly agree
  - Agree
  - Neutral
  - Disagree
  - Strongly disagree
  - Never discussed
- My mentor was able to provide adequate advice/feedback about family planning
  - Strongly agree
  - Agree
  - Neutral
  - Disagree
  - Strongly disagree
  - Never discussed
- My mentor was able to provide adequate advice/feedback about work-life balance
  - Strongly agree
  - Agree
  - Neutral
  - Disagree
  - Strongly disagree
  - Never discussed
- My mentor was able to provide adequate advice/feedback about financial planning
  - Strongly agree
  - Agree
  - Neutral
  - Disagree
  - Strongly disagree
  - Never discussed
- In your words, what was the most meaningful contribution from your mentor
- In your words, what is something you wished your mentor could have done/aided you with?
- It is important to have a mentor when pursuing plastic surgery
  - Strongly agree
  - Agree
  - Neutral
  - Disagree
  - Strongly disagree
- It is important for female plastic surgeons to have female mentors
  - Strongly agree
  - Agree
  - Neutral
  - Disagree
  - Strongly disagree
- It is important for a mentee to share the same race/ethnicity as their mentors
  - Strongly agree
  - Agree
  - Neutral
  - Disagree
  - Strongly disagree
- Having a mentor in plastic surgery will better prepare me for a career in plastic surgery
  - Strongly agree
  - Agree
  - Neutral
  - Disagree
  - Strongly disagree
